# Magnetic resonance imaging scoring system of the lower limbs in adult patients with suspected idiopathic inflammatory myopathy

**DOI:** 10.1007/s10072-024-07386-y

**Published:** 2024-02-21

**Authors:** Laura Ludovica Gramegna, Rita Rinaldi, Laura Maria Beatrice Belotti, Luca Vignatelli, Giovanni Sighinolfi, Valentina Papa, Roberta Costa, Roberto D’Angelo, Claudio Bianchini, Claudio Graziano, Lorenzo Cirignotta, Rita Mule, David Neil Manners, Caterina Tonon, Giovanna Cenacchi, Raffaele Lodi

**Affiliations:** 1 Programma Di Neuroimmagini Funzionali E Molecolari, IRCCS Istituto Delle Scienze Neurologiche Di Bologna, Bologna, Italy; 2 Department of Biomedical and Neuromotor Sciences (DIBINEM), University of Bologna, Bologna, Italy; 3 Clinica Neurologica Rete Neurologica Metropolitana, Sede Neurologia Policlinico S. Orsola, IRCCS Istituto Delle Scienze Neurologiche Di Bologna, Bologna, Italy; 4 Epidemiology and Statistics Unit, IRCCS, Istituto Delle Scienze Neurologiche Di Bologna, Bologna, Italy; 5 IRCCS Istituto Delle Scienze Neurologiche Di Bologna, Bologna, Italy; 6 U.O. Genetica Medica, AUSL Della Romagna, Cesena, Italy; 7 IRCCS Azienda Ospedaliero-Universitaria Di Bologna, Policlinico Di Sant’Orsola, UO Reumatologia, Bologna, Italy

**Keywords:** Myositis, Muscular diseases, Edema, Diagnosis

## Abstract

**Purpose:**

We aim to propose a visual quantitative score for muscle edema in lower limb MRI to contribute to the diagnosis of idiopathic inflammatory myopathy (IIM).

**Material and methods:**

We retrospectively evaluated 85 consecutive patients (mean age 57.4 ± 13.9 years; 56.5% female) with suspected IIM (muscle weakness and/or persistent hyper-CPK-emia with/without myalgia) who underwent MRI of lower limbs using T2-weighted fast recovery-fast spin echo images and fat-sat T2 echo planar images. Muscle inflammation was evaluated bilaterally in 11 muscles of the thigh and eight muscles of the leg. Edema in each muscle was graded according to a four-point Likert-type scale adding up to 114 points ([11 + 8)] × 3 × 2). Diagnostic accuracy of the total edema score was explored by assessing sensitivity and specificity using the area under the ROC curve. Final diagnoses were made by a multidisciplinary Expert Consensus Panel applying the Bohan and Peter diagnostic criteria whenever possible.

**Results:**

Of the 85 included patients, 34 (40%) received a final diagnosis of IIM (IIM group) while 51 (60%) received an alternative diagnosis (non-IIM group). A cutoff score ≥ 18 was able to correctly classify patients having an IIM with an area under the curve of 0.85, specificity of 96%, and sensitivity of 52.9%.

**Conclusion:**

Our study demonstrates that a quantitative MRI score for muscle edema in the lower limbs (thighs and legs) aids in distinguishing IIM from conditions that mimic it.

**Supplementary Information:**

The online version contains supplementary material available at 10.1007/s10072-024-07386-y.

## Introduction

Idiopathic inflammatory myopathies (IIMs) are a heterogeneous group of diseases characterized by progressive and symmetric proximal muscle weakness, elevated serum levels of skeletal muscle enzymes (e.g., creatine phosphokinase (CPK)), presence of specific autoantibodies, electromyography changes, and primary inflammatory infiltration in muscle biopsy [1, 2].

The diagnosis of IIM can be challenging in clinical practice as several conditions mimic it, including muscular dystrophies, metabolic myopathies, endocrine or toxic myopathies, and systemic inflammatory diseases [3]. The European League Against Rheumatism (EULAR) and the American College of Rheumatology (ACR) have recently proposed EULAR-ACR classification criteria to distinguish IIMs from mimics using 16 clinical and readily available laboratory/histopathological features [3]. Two models, with or without muscle biopsy results, were developed and according to this consensus, a diagnosis of definite, probable, or possible IIM can be obtained based on total scores. However, there are several limitations to the EULAR-ACR Criteria Project, such as exclusion of normal controls from the external validation cohort, data missing in the derivation data set, and exclusion of validation samples and MRI data [3].

MRI has several advantages in clinical practice, including detection of muscle edema and fatty replacement using T2 weighted and short tau inversion recovery (STIR) [4]. Previous exploratory muscle MRI studies correlated muscle edema with clinical markers of severity [4, 5] in heterogeneous subgroups of IIM patients; however, a universally accepted qualitative and quantitative MRI scoring system for patients with IIMs is lacking. Most studies performed MRI on bilateral thigh muscle alone [4, 6–10] and used binomial classification (present/absent) or qualitative visual scoring methods of muscle edema (Table 1 supplementary material). Only recently there was an attempt to obtain a cutoff value that distinguishes IIMs from other muscular dystrophy [10].

Distinguish IIM from other mimics is of great importance in clinical practice as IIM requires immunosuppressive therapy, which can be dangerous in other types of muscle diseases with remarkably similar presentation [11].

The aims of this study were to assess the potential role for whole lower limb muscles MRI in the diagnosis of IIMs through a newly devised edema total scoring system, to describe its feasibility in clinical practice, and to explore its diagnostic accuracy to distinguish between patients with diagnoses of IIM and mimics.

## Materials and methods

### Study design and patient selection

**Fig. 1:**
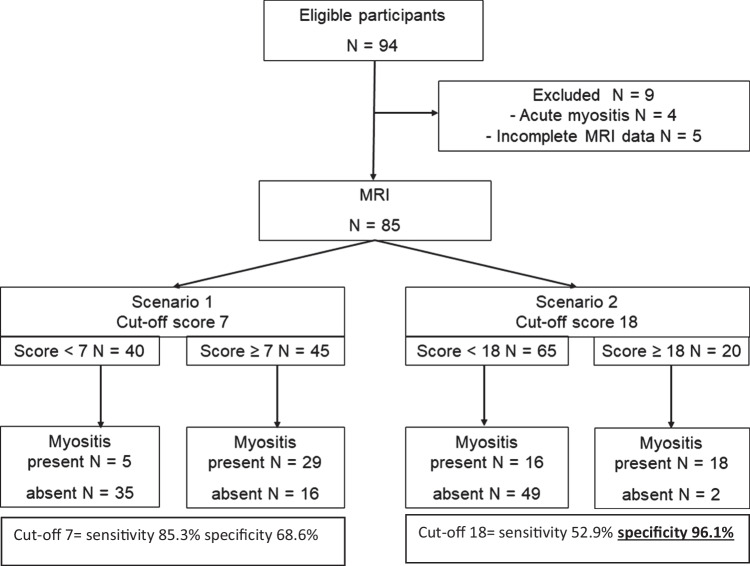
Flow diagram of eligible and included participants and edema scores. Patients were classified according to the two scenarios of the MRI edema scoring system (Scenario 1, cutoff score = 7, and Scenario 2, cutoff score = 18). Specifically, considering 7 as the cutoff score (Scenario 1), 40 patients had score < 7 (negative MRI and among them 5 patients had a definitive diagnosis of IIM) and 45 patients had a score ≥ 7 (positive MRI and among them 29 patients had a definitive diagnosis of IIM. Considering 18 as the cutoff score (Scenario 2), 65 patients had score < 18 (negative MRI and among them 16 had a definitive diagnosis of IIM) and 20 patients had score ≥ 18 (positive MRI and among them 18 had a final diagnosis of definitive myositis, IIM)

This was a retrospective cohort study of patients evaluated between January 2008 and September 2018 in the “Diagnostic Therapeutic Assistance Path (PDTA) for adult muscle diseases” of the Policlinico Sant'Orsola-Malpighi, Bologna, Italy. Inclusion criteria were adults (age ≥ 18 years) with suspected inflammatory myopathy, i.e., muscle weakness and/or persistent hyper-CPK-emia with/without myalgia (Fig. 1). Patients with a clear non-inflammatory disease (i.e., clear hereditary or metabolic myopathy) were excluded.

The study was approved by local IRB Ethic Committee (CE AVEC 850–2021-OSS-AUSLBO), and informed consent was waived due to the retrospective nature of the study.

### Diagnostic evaluation pathway

All patients underwent neurological and rheumatological evaluations to assess muscle weakness, and multisystemic involvement such as skin rashes and esophageal or pulmonary dysfunction, needle electromyography with spontaneous muscle activity and quantitative Motor Units study, assays for CPK, lactate dehydrogenase (LDH), transaminase, myositis-specific and myositis-associated antibodies. A muscle biopsy of the vastus lateralis to assess skeletal muscle inflammation and/or other changes was proposed to all patients. Steroids were administered to all patients with definitive diagnoses or when IIM was not diagnosed by laboratory or histopathological testing, but symptoms worsened as *ex juvantibus* criteria.

### Histological evaluation

Muscle samples were snap-frozen, cross-sectioned, and stained using a panel of routine histochemical methods: hematoxilyn/eosin, modified Gomori trichrome, reduced nicotinamide adenine dinucleotide tetrazolium reductase (NADH-TR), combined cytochrome oxidase (COX) and succinate dehydrogenase (SDH), adenosine triphosphatases (ATPases), and acid and alkaline phosphatases [12]. Muscle cross sections were processed by immunohistochemistry using mouse monoclonal antibodies to major histocompatibility complex class I (MHC-I) and neonatal myosin (MHC-n) to check regeneration rate, as previously described [13]. Small samples from each biopsy were fixed in 2.5% glutaraldehyde in cacodylate buffer, post-fixed in 1% OsO_4_, dehydrated, and embedded in araldite. These sections were stained with uranyl acetate and lead citrate and were observed on a CM100 Transmission Electron Microscope [13]. Stained muscle sections were observed by an experienced histopathologist with more than 20 years of experience in muscular disorders (G.C.) to check for morphological alterations suggestive of inflammatory myopathy.

### MRI evaluation

MRI of the upper and lower limbs was performed in all patients. All images were acquired using a 1.5-T (GE Medical Systems Signa HDx) MRI scanner at the Functional Unit of the Department of Biomedical and Neuromotor Sciences at the University of Bologna. Imaging was performed in the axial plane using T2-weighted sequences. Excitation and signal acquisition were acquired using an 8-channel phased-array coil. Initial scanning with sagittal and coronal views was performed for localization. Images were obtained of both the thighs and legs. The following pulse sequences were used: T2-weighted bi-dimensional axial fast recovery-fast spin echo (FR-FSE) images were acquired (echo time (TE) = 85 ms, repetition time (TR) = 14,080 ms, field of view (FOV) = 34 cm); fat-sat T2-weighted axial echo planar imaging (EPI) images were acquired (TE = 30, 60, 90 ms; TR = 10,000 ms; FOV = 34 cm). Slice thickness: 5 mm, gap 1 mm. Acquisition time was 20 min.

**Fig. 2:**
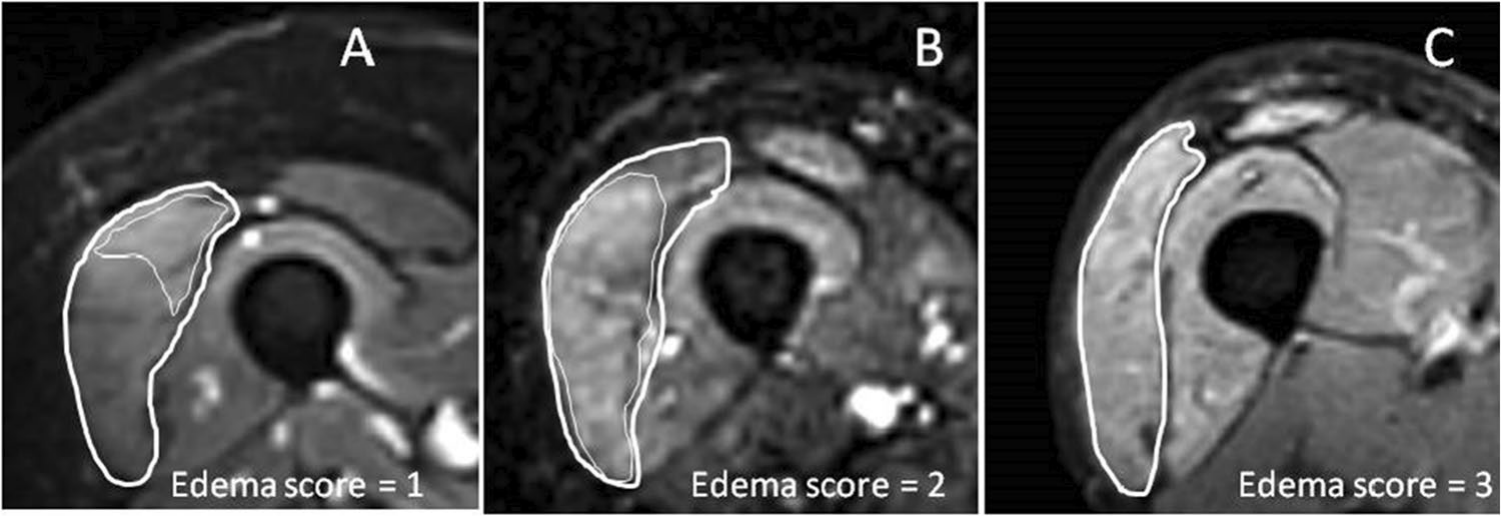
Axial EPI-T2-weighted sequences (TE = 90 ms) of the thigh depicting three different grades of edema in the right vastus lateralis muscle. **A** Edema was scored as 1 because of the slight involvement of 1/3 of muscle; the patient was negative for IIM and had a final diagnosis of FSHD genetically confirmed. **B** Edema was scored as 2 because it was moderated and involved 2/3 of the muscle area; the patient had a final diagnosis IIM. **C** Edema was scored as 3 since it involved the total muscle area; the patient had a final diagnosis of IIM

The MR images generated were reviewed by two neuroradiologists with > 20 (R.L.) and > 10 years (L.L.G.) of experience in the field of neuromuscular disorders. Based on visual inspection of the axial FR-FSE and EPI-T2 weighted sequence, the edema of each muscle was graded according to a four-point scale (0 = no edema; 1 = slight edema involving 1/3 of muscle area and/or being of slight hyperintensity; 2 = moderate edema involving 2/3 of muscle area and/or being of moderate hyperintensity; or 3 = severe edema involving total muscle area and/or being of severe hyperintensity) (Fig. 2). The presence of fibro-adipose tissue within the muscle was evaluated with the same four-point scale.

Muscle inflammation was evaluated in 11 individual muscles of the thighs (vastus lateralis, vastus intermedius, rectus femoris, vastus medialis, sartorius, adductor longus, adductor magnus, gracilis, biceps femoris, semitendinosus, semimembranosus) and 8 individual muscles of the legs (tibialis anterior, extensor digitorum longus, peroneus, tibialis posterior, flexor digitorum longus, soleus, gastrocnemius (caput lateralis), gastrocnemius (caput medialis)). Therefore, the total highest possible score was 114, i.e., ([11 + 8] × 3 points × 2 legs).

The neuroradiologists evaluated the images independently and blinded to clinical information. Divergent scores for each muscle were discussed and final judgment was reached by consensus, with these scores used for computing the final cutoff score.

### Reference standard for the final diagnosis

The reference standard was the diagnostic decision established using a consensus among a multidisciplinary team [14] consisting of two neurologists, one rheumatologist, and one histopathologist, evaluating clinical, neurophysiological, laboratory, and histopathological findings. Radiological images were excluded from the procedure. Whenever possible, diagnosis of IIM was based on Bohan and Peter diagnostic criteria [15, 16]. The diagnosis of idiopathic hyper-CPK-emia was made as previously described by Kyriakides and colleagues [17–19]. In cases of patients refusing biopsy, other information was used to reach a consensus diagnosis on a single-patient basis. The final diagnostic groups were “IIM” and “non-IIM.” EULAR-ACR [3] scores were assigned to all patients.

### Statistical analysis

Continuous variables were expressed as mean and standard deviation (SD), while categorical variables were expressed by absolute numbers (*n*) and percentages (%). Groups were compared using Student’s *t*-test or Mann–Whitney *U* test, as appropriate, for continuous variables, and Pearson chi-square test or Fisher’s exact test, as appropriate, for categorical variables. The Shapiro–Wilk test was employed to evaluate the normality of the data.

For radiological evaluation, the intraclass correlation coefficient (ICC) was used to measure inter-rater agreement. A two-way mixed-effects model was applied, and the single-rater absolute agreement was evaluated [20]. Values < 0.5 indicate poor reliability [20].

Diagnostic accuracy of the MRI edema total scoring system versus the reference standard was explored measuring sensitivity, specificity, and the area under the ROC curve. Optimal sensitivity and specificity cutoffs were explored with both a statistical method (Youden test) and a clinical scenario method (maximization of post-test probability for negative test and for positive test). The variability of estimates was expressed for each measure with 95% confidence interval (95% CI). Statistical analysis was performed using the Stata SE, 14.2 statistical package (Stata Corp.). To explore whether it would be possible to construct a simplified index, ROC analysis was also performed on supplementary scores constructed by eliminating muscles from the total if they demonstrated low inter-rater agreement (i.e., < 0.5).

In the group of patients with IIM diagnosis, age was correlated with both the total edema score and total fibro-adipose tissue score using the Spearman correlation test.

## Results

### Patient characteristics

A total of 85 patients (mean age 57.4 ± 13.9 years; 48 females) were included in the study.

Among them, 34 (40.0%; mean age 61.2 ± 12.6 years; 22 females) had a definitive diagnosis of IIM according to the reference standard. The remaining 51 (60.0%; mean age 55.0 ± 14.3 years; 26 females) received an alternative diagnosis (non-IIM group), including 30(58.8%%s) patients with idiopathic hyper-CPK-emia, six (11.7%) with undetermined myopathy, five (9.8%) with genetic myopathy (in particular one patient received a genetic diagnosis of calpainopathy, one of fascioscapulohumeral muscular dystrophy, and in three, the diagnosis was made based on histopathologic findings consistent with dystrophynopathy), three (5.9%) with mitochondrial myopathy, two (3.9%) with statin-induced myopathy, two (3.9%) with endocrine myopathy, one (2.0%) with steroid-induced myopathy, one (2.0%) with antineutrophilic cytoplasmic antibody (cytoplasmic type) (c-ANCA) vasculitis, and one (2.0%) with multiple radiculopathy (lower limbs). Fourteen patients with IIM out of 85 (16.5%) patients were undergoing anti-immunosuppressant therapy at the time of the MRI examination.

Table 1 describes IIM versus non-IIM patients.

**Table 1:** Characterization of IIM versus non-IMM patients

	IIM patients (*N* = 34)	Non-IMM patients (*N* = 5 1)	*p* value
Age (years)	61.18 ± 12.61	54.96 ± 14.35	0.0434
Sex (female) (*n*, %)	22 (64.7%)	26 (51.0%)	0.2111
Diagnosis			
IIM	34 (100.0%)	—	—
Idiopathic hyper-CPK-emia (*)	—	30 (58.8%)	—
Undetermined myopathy	—	6 (11.7%)	—
Genetic myopathy		5 (9.8%)	
Mitochondrial myopathy	—	3 (5.9%)	—
Statin-induced myopathy	—	2 (3.9%)	—
Endocrine myopathy	—	2 (3.9%)	—
Steroid-induced myopathy	—	1 (2.0%)	—
Vasculitis c-ANCA	—	1 (2.0%)	—
Multiple radiculopathy (lower limbs)	—	1 (2.0%)	—
EULAR score	70.71 ± 34.15	16.23 ± 21.31	< 0.001
Muscle manifestation			
Symmetric hyposthenia (upper limbs)	19 (55.9%)	4 (7.8%)	< 0.001
Symmetric hyposthenia (lower limbs)	26 (76.5%)	9 (17.6%)	< 0.001
Neck: flexor > extensor	11 (32.4%)	3 (5.9%)	0.002
Lower limbs: proximal > distal	26 (76.7)	8 (15.7%)	< 0.001
Skin manifestation			
Heliotrope rash	5 (14.7%)	0 (0.0%)	0.008
Gottron papules	4 (11.8%)	0 (0.0%)	0.023
Gottron sign	6 (17.6%)	0 (0.0%)	0.003
Esophageal motility disorder	7 (20.6%)	2 (3.9%)	0.026
Anti Jo1	3 (8.8%)	0 (0.0%)	0.061
Elevated level of CPK, LDH, transaminase	30 (88.2%)	46 (90.2%)	0.999
Muscle biopsy	28 (82.4%)	21 (41.2%)	< 0.001
Inflammatory changes	21 (77.8%)	3 (15%)	< 0.001
Total edema score	21.80 ± 16.9	5.40 ± 6.30	< 0.001
Thigh edema	12.29 ± 12.69	1.49 ± 3.26	< 0.001
Leg edema	9.79 ± 6.28	4.00 ± 4.25	< 0.001
Total fibro-adipose tissue score	7.85 ± 16.46	7.00 ± 14.71	0.7866
Thigh fibro-adipose tissue	5.41 ± 11.68	4.82 ± 10.91	0.8128

Data are presented as mean ± standard deviation, SD, or number, *n* (%)

*IIM* idiopathic inflammatory myopathy, *CPK* creatine phosphokinase, *EULAR* European League Against Rheumatism, *c-ANCA* antineutrophilic cytoplasmic antibody (cytoplasmic type), *LDH* lactate dehydrogenase, *Anti Jo1* auto-antibodies to histidyl tRNA synthetase

^*^As described by Kyriakides et al. [18, 19]

Patients in the IIM group were more likely than the non-IIM group to present with neck muscles hyposthenia flexor > extensor (32.4% (11/34) vs. 5.9% (3/51), respectively; *p* = 0.002), symmetric upper limb hyposthenia (55.9% (19/34) vs. 7.8% (4/51); *p* < 0.001), symmetric lower limb hyposthenia (76.5% (26/34) vs. 17.6% (9/51); *p* < 0.001), proximal > distal (76.5% (26/34) vs. 15.7% (8/51); *p* < 0.001). No patients in the non-IIM group presented with skin alterations, while 44.1% (15/34) of the IIM group had dermatologic manifestations. Patients in the IIM group had a higher rate of esophageal motility disorders than the non-IIM group (20.6% (7/34) vs. 3.9% (2/51), respectively; *p* = 0.026). Elevated levels of CPK, LDH, and transaminase did not differ between the two groups. The mean EULAR-ACR score was higher for the IIM group (70.7 ± 34.2) compared to the non-IIM group (16.2 ± 21.3) (*p* < 0.001).

### Histological evaluation

Only 49/85 (57.6%) patients consented to muscle biopsy. Among them, 57.1% (28/49) received a final diagnosis of IIM, while 42.9% (21/49) had IIM mimics. In the IIM group, inflammatory changes were present in 21/28 patients (77.8%) and in the non-IIM group in 3/21 patients (15%).

In seven IIM patients, the muscle biopsy was reported as negative since it showed very few myopathic changes; 3/7 showed fibro-fatty substitution (patient 73, 30, and 69), 2/7 (69 and 63) showed moth-eaten fibers, and six patients had sarcoplasmic expression of MHC-I below the 50% cutoff [13]. In five of these patients, the diagnosis was made due to a strong response to immunotherapy (i.e., an ex adjuvantibus diagnosis). In one patient, the diagnosis was based on clear symptoms, including a cutaneous rash, and laboratory analysis showing anti-Jo positivity. In one patient, the biopsy showed no inflammatory changes but tested positive for anti-SR1, which is compatible with inflammatory necrotizing myopathy.

In 3 non-IIM patients (patients 34, 36, and 59), the muscle biopsy was reported to have inflammatory signs; 2/3 had myophagy and inflammatory cells (patients 34 and 59), and 1/3 (patient 36) showed rimmed vacuoles and intranuclear filaments at the ultrastructural level. No specific differences were found in comparison to the biopsies that tested positive in patients with definitive IIM diagnosis. In two of these cases, a final diagnosis of genetic myopathy was established, one being calpainopathy and the other one a dystrophy due to a mutation in the Titin gene. In the third patient, a final diagnosis of undetermined myopathy was made.

### MRI evaluation

The overall inter-rater agreement, considering both the thighs and legs, was excellent (ICC = 0.92 (95% CI 0.87–0.95)), as was the agreement for the thighs (ICC = 0.92 (95% CI 0.88–0.95)), and the legs (ICC = 0.84 (95% CI 0.74–0.90)). ICC values for the single muscles can be found in Table 2.

**Table 2:** Evaluation of the intra-class correlation coefficient (ICC) between the two readers

Total edema score: ICC = 0.92 (0.87–0.95)					
Upper leg score		0.92 (0.88–0.95)	Lower leg score		0.84 (0.74–0.90)
Vastus lateralis	Left	0.89 (0.84–0.93)	Tibialis anterior	Left	0.80 (0.71–0.86)
	Right	0.84 (0.77–0.90)		Right	0.83 (0.75–0.89)
	Bilateral	0.91 (0.86–0.94)		Bilateral	0.84 (0.76–0.89)
Vastus intermedius	Left	0.95 (0.92–0.97)	Extensor digitorum longus	Left	0.77 (0.67–0.85)
	Right	0.87 (0.81- 0.92)		Right	0.55 (0.34–0.69)
	Bilateral	0.94 (0.91–0.96)		Bilateral	0.71 (0.54–0.82)
Rectus femoris	Left	0.87 (0.81–0.91)	Peroneus	Left	0.60 (0.45–0.72)
	Right	0.84 (0.76–0.89)		Right	0.58 (0.42–0.70)
	Bilateral	0.86 (0.79–0.91)		Bilateral	0.65 (0.50–0.75)
Vastus medialis	Left	0.87 (0.81–0.91)	Tibialis posterior	Left	0.72 (0.60–0.81)
	Right	0.85 (0.78–0.90)		Right	0.76 (0.65–0.84)
	Bilateral	0.87 (0.80–0.91)		Bilateral	0.84 (0.77–0.90)
Sartorius	Left	0.40 (0.21–0.57)	Flexor digitorum longus	Left	0.62 (0.47–0.73)
	Right	0.45 (0.26–0.60)		Right	0.64 (0.50–0.75)
	Bilateral	0.49 (0.31–0.64)		Bilateral	0.77 (0.66–0.84)
Adductor longus	Left	0.89 (0.84–0.93)	Soleus	Left	0.51 (0.33–0.66)
	Right	0.79 (0.70–0.86)		Right	0.66 (0.47–0.78)
	Bilateral	0.87 (0.81–0.92)		Bilateral	0.64 (0.44–0.77)
Adductor magnus	Left	0.82 (0.74–0.88)	Gastrocnemius (caput lateralis)	Left	0.50 (0.31–0.64)
	Right	0.88 (0.83–0.92)		Right	0.69 (0.56–0.78)
	Bilateral	0.89 (0.83–0.92)		Bilateral	0.70 (0.57–0.79)
Gracilis	Left	0.35 (0.15–0.52)	Gastrocnemius (caput medialis)	Left	0.57 (0.41–0.70)
	Right	0.48 (0.31–0.63)		Right	0.73 (0.62–0.82)
	Bilateral	0.48 (0.29–0.63)		Bilateral	0.72 (0.59–0.81)
Biceps femoris	Left	0.74 (0.62–0.82)			
	Right	0.80 (0.71–0.87)			
	Bilateral	0.78 (0.68–0.85)			
Semitendinosus	Left	0.69 (0.56–0.79)			
	Right	0.76 (0.65–0.84)			
	Bilateral	0.78 (0.68–0.85)			
Semimembranosus	Left	0.83 (0.75–0.89)			
	Right	0.74 (0.63–0.83)			
	Bilateral	0.85 (0.78–0.90)			

In the group of patients with diagnoses of IIM, the average total edema score was 21.79 ± 16.95, specifically 12.29 ± 12.69 in the thighs and 9.50 ± 6.41 in the legs. Table 3 includes details regarding the involvement of each muscle.

**Table 3:** Evaluation of the average edema score and the single muscle edema score in the IIM and IIM mimics patients

	IIM (*N* = 34)	Non-IIM (*N* = 51)
Total edema score	21.79 ± 16.95 33 pts (97.1%)	(5.43 ± 6.28)—33 (64.7%)
Upper leg edema score	(12.29 ± 12.69)—27 (79.4%)	(1.43 ± 3.21)—14 (27.5%)
Lower leg edema score	(9.50 ± 6.41)—32 (94.1%)	(4.00 ± 4.25)—32 (62.7%)
Single muscle upper leg		
Vastus lateralis	2 [3]—24 (70.6%)	0 [0]—9 (17.6%)
Vastus intermedius	1.5 [2.75]—20 (58.8%)	0 [0]—5 (9.8%)
Rectus femoris	1 [3]—18 (52.9%)	0 [0]—4 (7.8%)
Vastus medialis	0.5 [2]—17 (50.0%)	0 [0]—2 (3.9%)
Sartorius	0 [1]—10 (29.4%)	0 [0]—3 (5.9%)
Adductor longus	0 [1]—10 (29.4%)	0 [0] – 0
Adductor magnus	0.5 [2]—17 (50.0%)	0 [0]—5 (9.8%)
Gracilis	0 [0]—7 (20.6%)	0 [0]—3 (5.9%)
Biceps femoris	0 [2]—15 (44.1%)	0 [0]—7 (13.7%)
Semitendinosus	0 [0]—8 (23.5%)	0 [0]—3 (5.9%)
Semimembranosus	0 [2]—14 (41.2%)	0 [0]—5 (9.8%)
Single muscle lower leg		
Tibialis anterior	1.5 [2]—20 (58.8%)	0 [0.5]—13 (25.5%)
Extensor digitorum longus	1 [2]—19 (55.9%)	0 [0]—9 (17.6%)
Peroneus	0 [2]—13 (38.2%)	0 [0]—8 (15.7%)
Tibialis posterior	0 [1]—10 (29.4%)	0 [0]—2 (3.9%)
Flexor digitorum longus	0 [0]—7 (20.6%)	0 [0]—2 (3.9%)
Soleus	1 [2]—18 (52.9%)	0 [1]—17 (33.3%)
Gastrocnemius (caput lateralis)	2 [1]—28 (85.3%)	1 [1]—27 (52.9%)
Gastrocnemius (caput medialis)	2 [1]—29 (85.3%)	1 [2]—28 (54.9%)

Data are mean ± SD, number of patients with a score > 0 (%), or median [IQR], number of patients with a score > 0 (%)

*IIM* idiopathic inflammatory myopathy

In patients with final diagnoses of IIM mimics, the average total edema score was 5.43 ± 6.28, specifically 1.43 ± 3.21 in the thighs and 4.00 ± 4.25 in the legs. Table 3 includes details about each muscle involvement.

Total bilateral lower limb edema, thigh bilateral edema, and leg bilateral edema scores were significantly higher in the IIM group than the non-IIM group (*p* < 0.001). The total burden of fibroadipose tissue, both in the thighs and legs, was not significantly different between groups (*p* = 0.7866 and *p* = 0.8128, respectively) (Table 1).

In the group patients with IIM diagnosis, there was no correlation between age and the edema scores (rho =  − 0.036, *p* = 0.841), and the association between age and fibro-adipose (FA) score was not significant (rho = 0.308 and 0.077).

There were no differences in the edema score in IIM patients between those receiving treatment (14/34) and those untreated (20/34) at the time of the MRI (23.6 ± 18.9 versus 20.6 ± 15.7, respectively).

### Diagnostic accuracy analysis

**Fig. 3:**
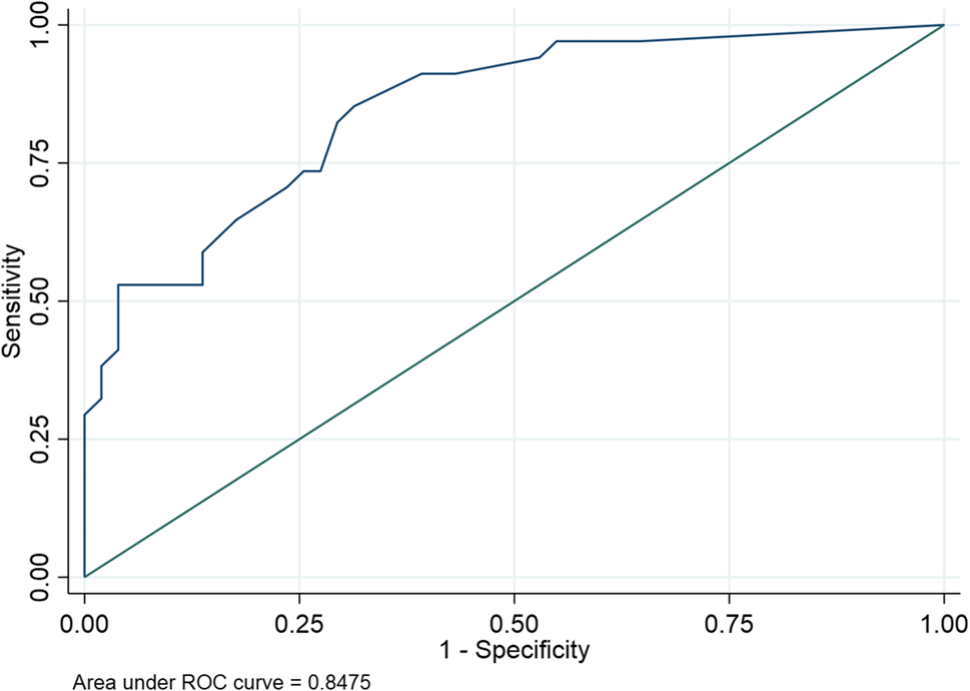
ROC curve. An exploratory diagnostic accuracy analysis of the edema scoring system showed an area under the ROC curve of 0.85 [95% CI 0.77–0.93]. To maximize specificity, the optimal cutoff score was ≥ 18. In this case, sensitivity was 52.9% (95% CI 35.1–70.2%) and specificity 96.1% (95% CI 86.5–99.5%); 78.8% of patients were correctly classified. To maximize sensitivity (corresponding to the best Youden’s Index, 53.9%), the optimal cutoff score was ≥ 7. In this case, sensitivity was 85.3% (95% CI 68.9–95%) and specificity 68.6% (95% CI 54.1–80.9%)

Exploratory diagnostic accuracy analysis of the edema total scoring system showed an area under the ROC curve of 0.85 (95% CI 0.77–0.93) (Fig. 3).

In Fig. 1, we reported the diagnostic accuracy values of different cutoff scores. To maximize specificity, the optimal cutoff score was ≥ 18. In this case, sensitivity was 52.9% (95% CI 35.1–70.2%) and specificity was 96.1% (95% CI 86.5–99.5%); 78.8% of patients were correctly classified. Due to the observed prevalence of 40%, the positive predictive value was 90.0%, and the negative predictive value was 75.4%. To maximize sensitivity (corresponding to the best Youden’s Index, 53.9%), the optimal cutoff score was ≥ 7. In this case, sensitivity was 85.3% (95% CI 68.9–95%) and specificity was 68.6% (95% CI 54.1–80.9%). The positive predictive value was 64.4%, and the negative predictive value was 87.5% (Fig. 1).

## Discussion

It is well known that clinicians face challenges in discriminating between IIM and its mimics as IIM may have a heterogenous presentation and course, and there is no single feature that could serve as a “gold standard” for diagnosis and/or classification [21]. Most importantly, if mimicking conditions are mistaken for autoimmune conditions in clinical practice, this misdiagnosis can lead to inappropriate and potential harmful immunosuppressive therapy [11].

In this study, we propose an imaging score that is easily applicable in clinical practice, as the MRI acquisition parameters are standard, and it only requires visual inspection of the lower limbs to assess the degree of edema in each muscle in the thighs and legs, ranging from 0 (absent) to 3 (severe). This score enables the comprehensive assessment of edema burden in the lower limbs and reveals that a cutoff score of 17 aids in distinguishing between IIM patients and mimics, with an AUC of 0.85, specificity of 96.1%, and sensitivity of 52.9%.

Our scoring system assessing the extent of edema demonstrates high reproducibility, as evidenced by excellent inter-rater agreement for both thighs and legs. This aspect can also be attributed to the semi-quantitative approach to assess muscle edema involvement. In particular, in our study, we introduced a discrete evaluation score for each muscle, whereas most previous studies either employed a binary classification (present or absent) for each muscle or focused solely on the thigh [10]. In this study, our goal was to determine the optimal cutoff score for this new standardized muscle edema score on MRI in distinguishing it from all other mimics. We have decided to recommend a cutoff of 18 in clinical practice since it yielded the highest specificity at 96.1%, even though it came with a suboptimal sensitivity of 52%. This suggests that our score should be utilized as a secondary tool to confirm a suspected diagnosis (see Scenario 2 in Fig. 3). In our study, we found no difference in the fibro-adipose score between patients with idiopathic inflammatory myopathies (IIM) and those without, suggesting that in the diagnostic process for IIM, edema is a more reliable indicator of the disease than fibro-adipose (FA) infiltration, even though FA may be present.

Relatively recently, the EULAR-ACR Classification Criteria have been proposed as a method for classifying adult and juvenile IIM to be used in clinical trials for myositis [3]. However, they present some limitations in clinical practice since the output consists of the definite, probable, and possible likelihood of having IIM. For the purpose of our analysis, we needed to establish a dichotomous classification of IIM versus non-IIM. The best cutoff point for EULAR criteria is still under debate since, after the publication of the original EULAR criteria, several studies have tried to validate those criteria in external derivation cohorts and have found different cutoff points in each population [22]. One study attempted to add MRI as a covariate in the original score and found that doing so (AUC = 0.86) was more likely to correctly diagnose IIM than the EULAR score alone (AUC = 0.80) [23]. However, the authors reported that the MRI was evaluated by the reporting radiologist using a binary score reflecting the presence or absence of muscle edema, there was no description of the muscles evaluated, and the authors did not specify the degree of edema or extent of muscle involvement [23]. In our study, we proposed a discrete evaluation score for each muscle whereas the majority of previous studies used a binomial classification for each muscle (present or absent) or evaluated only the thigh [10].

In our study, two non-IIM patients with definitive diagnosis of genetic dystrophy had muscle biopsies reported to show inflammatory signs, with no specific differences compared to biopsies that tested positive in patients with definitive diagnoses. This is not surprising, as it is well-known that an inflammatory pattern can also be observed in muscle biopsies of these diseases [24].

Our study presents several limitations, primarily related to the relatively small sample size and its retrospective nature. However, our results may have significant implications as our proposed cutoff score of 18 has a higher specificity (96%) in comparison to the EULAR criteria (88% with biopsy and 82% without), and we suggest that in selected case, MRI could confirm the diagnosis, avoiding biopsy and could be considered for future revision of the EULAR criteria. Our patients exhibited variability in their therapy, as 14 out of 85 (16.5%) patients were undergoing anti-immunosuppressant therapy at the time of the MRI examination. The available data seem to support that MRI results are not affected. In our study, there were no differences in the edema score in IIM patients between those receiving treatment (14/34) and those untreated (20/34) at the time of the MRI (23.6 ± 18.9 versus 20.6 ± 15.7, respectively).

In conclusion, our study demonstrates that a quantitative MRI score for muscle edema in the lower limbs (thighs and legs) aids in distinguishing IIM from conditions that mimic it. This lends weight to the idea that, especially in the initial stages, edema is a more reliable indicator of the disease than fibro-adipose (FA) infiltration, even when FA is present. Further studies are needed to test whether MRI could be also a good outcome measure.

### Supplementary Information

Below is the link to the electronic supplementary material.

Supplementary file1 (DOCX 16 KB)

## Data Availability

The data supporting the findings of this study are available from the corresponding author upon reasonable request.

## References

[1] Dalakas MC (2015). Inflammatory muscle diseases. N Engl J Med.

[2] Betteridge Z, Gunawardena H, North J, Slinn J, McHugh N (2007). Anti-synthetase syndrome: a new autoantibody to phenylalanyl transfer RNA synthetase (anti-Zo) associated with polymyositis and interstitial pneumonia. Rheumatology (Oxford).

[3] Lundberg IE, Tjarnlund A, Bottai M, Werth VP, Pilkington C, de Visser M, Alfredsson L, Amato AA, Barohn RJ, Liang MH, Singh JA, Aggarwal R, Arnardottir S, Chinoy H, Cooper RG, Danko K, Dimachkie MM, Feldman BM, Garcia-De La Torre I, Gordon P, Hayashi T, Katz JD, Kohsaka H, Lachenbruch PA, Lang BA, Li Y, Oddis CV, Olesinska M, Reed AM, Rutkowska-Sak L, Sanner H, Selva-O'Callaghan A, Song YW, Vencovsky J, Ytterberg SR, Miller FW, Rider LG, International Myositis Classification Criteria Project Consortium tER, the Juvenile Dermatomyositis Cohort Biomarker S, Repository (2017). 2017 European League Against Rheumatism/American College of Rheumatology Classification Criteria for Adult and Juvenile Idiopathic Inflammatory Myopathies and Their Major Subgroups. Arthritis Rheumatol.

[4] Tomasova Studynkova J, Charvat F, Jarosova K, Vencovsky J (2007). The role of MRI in the assessment of polymyositis and dermatomyositis. Rheumatology (Oxford).

[5] Malattia C, Damasio MB, Madeo A, Pistorio A, Providenti A, Pederzoli S, Viola S, Buoncompagni A, Mattiuz C, Beltramo A, Consolaro A, Ravelli A, Ruperto N, Picco P, Magnano GM, Martini A (2014). Whole-body MRI in the assessment of disease activity in juvenile dermatomyositis. Ann Rheum Dis.

[6] Pipitone N, Notarnicola A, Levrini G, Spaggiari L, Scardapane A, Iannone F, Lapadula G, Pattacini P, Zuccoli G, Salvarani C (2016). Do dermatomyositis and polymyositis affect similar thigh muscles? A comparative MRI-based study. Clin Exp Rheumatol.

[7] Davis WR, Halls JE, Offiah AC, Pilkington C, Owens CM, Rosendahl K (2011). Assessment of active inflammation in juvenile dermatomyositis: a novel magnetic resonance imaging-based scoring system. Rheumatology (Oxford).

[8] Andersson H, Kirkhus E, Garen T, Walle-Hansen R, Merckoll E, Molberg O (2017). Comparative analyses of muscle MRI and muscular function in anti-synthetase syndrome patients and matched controls: a cross-sectional study. Arthritis Res Ther.

[9] Zheng Y, Liu L, Wang L, Xiao J, Wang Z, Lv H, Zhang W, Yuan Y (2015). Magnetic resonance imaging changes of thigh muscles in myopathy with antibodies to signal recognition particle. Rheumatology (Oxford).

[10] Barsotti S, Mugellini B, Torri F, Minichilli F, Tripoli A, Cardelli C, Cioffi E, Zampa V, Siciliano G, Caramella D, Ricci G, Mosca M (2023). The role of magnetic resonance imaging in the diagnostic work-out of myopathies: differential diagnosis between inflammatory myopathies and muscular dystrophies. Clin Exp Rheumatol.

[11] Michelle EH, Mammen AL (2015). Myositis mimics. Curr Rheumatol Rep.

[12] Cenacchi G, Peterle E, Fanin M, Papa V, Salaroli R, Angelini C (2013). Ultrastructural changes in LGMD1F. Neuropathology.

[13] Salaroli R, Baldin E, Papa V, Rinaldi R, Tarantino L, De Giorgi LB, Fusconi M, Malavolta N, Meliconi R, D'Alessandro R, Cenacchi G (2012). Validity of internal expression of the major histocompatibility complex class I in the diagnosis of inflammatory myopathies. J Clin Pathol.

[14] Rutjes AW, Reitsma JB, Coomarasamy A, Khan KS, Bossuyt PM (2007) Evaluation of diagnostic tests when there is no gold standard. A review of methods. Health Technol Assess 11(50):iii, ix–51. 10.3310/hta11500 10.3310/hta11500 18021577

[15] Bohan A, Peter JB (1975). Polymyositis and dermatomyositis (first of two parts). N Engl J Med.

[16] Bohan A, Peter JB (1975). Polymyositis and dermatomyositis (second of two parts). N Engl J Med.

[17] Kyriakides T, Angelini C, Schaefer J, Mongini T, Siciliano G, Sacconi S, Joseph J, Burgunder JM, Bindoff LA, Vissing J, de Visser M, Hilton-Jones D (2013). EFNS review on the role of muscle biopsy in the investigation of myalgia. Eur J Neurol.

[18] Kyriakides T, Angelini C, Schaefer J, Sacconi S, Siciliano G, Vilchez JJ, Hilton-Jones D, Federation E, of Neurological S,  (2010). EFNS guidelines on the diagnostic approach to pauci- or asymptomatic hyperCKemia. Eur J Neurol.

[19] Kyriakides T, Angelini C, Vilchez J, Hilton-Jones D (2020). European Federation of the Neurological Societies guidelines on the diagnostic approach to paucisymptomatic or asymptomatic hyperCKemia. Muscle Nerve.

[20] Koo TK, Li MY (2016). A Guideline of Selecting and Reporting Intraclass Correlation Coefficients for Reliability Research. J Chiropr Med.

[21] Aggarwal R, Oddis CV, Goudeau D, Fertig N, Metes I, Stephens C, Qi Z, Koontz D, Levesque MC (2015). Anti-signal recognition particle autoantibody ELISA validation and clinical associations. Rheumatology (Oxford).

[22] Zoske J, Schneider U, Siegert E, Kleefeld F, Preusse C, Stenzel W, Hahn K (2021). Performance of ENMC and EULAR/ACR classification systems applied to a single tertiary center cohort of dermatomyositis patients. Neurol Res Pract.

[23] Luu Q, Day J, Hall A, Limaye V, Major G (2019). External validation and evaluation of adding MRI or extended myositis antibody panel to the 2017 EULAR/ACR Myositis Classification Criteria. ACR Open Rheumatol.

[24] Tidball JG, Welc SS, Wehling-Henricks M (2018). Immunobiology of inherited muscular dystrophies. Compr Physiol.

