# Supplementary material for: Magnetic resonance imaging scoring system of the lower limbs in adult patients with suspected idiopathic inflammatory myopathy
Source: Neurol Sci. 2024 Feb 21;45(7):3461–70. doi: 10.1007/s10072-024-07386-y (PMC11176218; doi:10.1007/s10072-024-07386-y)
Supplement: Supplementary file 1 — Supplementary file1 (DOCX 16 KB) [file 10072_2024_7386_MOESM1_ESM.docx]

Supplementary table 1.

| **Article** | **N** | **Disease** | **MRI location** | **Units of muscle evaluation (number of units)** | **MRI edema**  **scoring**  **(raw data)/MRI fatty deposition scoring** | **MRI edema**  **scoring (statistical)** | **Findings** |
| --- | --- | --- | --- | --- | --- | --- | --- |
| Pipitone 2016 [8] | 71 | PM, DM | Pelvic,  Thigh | Individual muscles (17) | B/-- | An MRI edema score (range 0-17) was calculated by adding the separate scores bilaterally and dividing them by two. | DM was more likely to affect anterior thigh muscles than PM |
| Barsotti 2016 [5] | 51 | PM (n=29), DM (n=22) | Thigh | Individual muscles (10):  RF, VM, VL, VI, sartorius, gracilis, AM, AL, BF, ST | PS (4)/B | An MRI edema score was calculated as the mean of the values in the different muscles on both sides | MRI edema score correlated with CPK and. PGA; with MMT when patients with *muscle fatty infiltration excluded* |
| Tomasova, Studynkova 200 [4]7 | 29 | PM (n=9), DM (n=20) | Thigh | Muscle groups (1) | C (10cm VAS)/-- | -- | Signal intensity of MRI is most closely correlated with disease activity, global clinical activity, and muscle disease activity evaluated semi-quantitatively using 10 cm VAS at acute presentation |
| Malattia 2014 [7] | 41 | JDM | Whole body | Muscle groups (42) | PS (3)/PS (3) | An MRI edema score was obtained as an arithmetical sum of all muscular group scores | Whole body MRI muscle scores correlated with MMT and CMAS |
| Davis 2011 [9] | 33 | JDM | Thigh | Muscle groups (4):  gluteal muscles, hamstrings,  quadriceps, and adductors | PS (4),  Soft-tissue edema (B),  Perifascicular edema (B)/-- | A total score of 20 was possible for each side | There was fair to moderate agreement between the two observers |
| Andersson 2017 [6] | 68 | ASS | Thigh | Anterior, posterior, and medial compartments (3) | PS (4) **extent** of edema,  PS (4) signal intensity of the edema ,  Fascial edema (B)/PS (6) | A total edema score consisting of 3 components—edema extent (18), edema intensity (18) and presence of fascial edema | The total edema score was associated with pathologic CK levels, anti-Ro52 and anti-Jo1 positivity |
| Zheng 2015 [10] | 12 | ASRP | Thigh | Individual muscles (12):  AL, AM, BF, gluteus maximus, BF (long head), gracilis, RF, sartorius, SM, ST, VI, VL, VM | PS (6)  The degree of edema (0-5 scale; from normal to moderate intrafascicular global edema changes)/PS (6) fatty infiltration according to the modified Mercuri scale (0-5 scale; from normal appearance to complete fatty infiltration) | MRI score ranges for fat from 0-120 and for edema from 0-12 | Fatty infiltration correlated with disease duration |
| Cox 2011 [11] | 32 | IBM | Whole body* | Individual muscles (68) | B/B | B | Number of muscles infiltrated by fat correlated with disease duration, MMT and serum creatinine kinase |
